# New Frontiers on Human Safe Insecticides and Fungicides: An Opinion on Trehalase Inhibitors

**DOI:** 10.3390/molecules25133013

**Published:** 2020-07-01

**Authors:** Camilla Matassini, Camilla Parmeggiani, Francesca Cardona

**Affiliations:** 1 Dipartimento di Chimica “Ugo Schiff”, Università degli Studi di Firenze, Via della Lastruccia 3-13, 50019 Sesto Fiorentino, Italy; camilla.parmeggiani@unifi.it; 2 European Laboratory for Non-linear Spectroscopy via Nello Carrara 1, 50019 Sesto Fiorentino, Italy

**Keywords:** antibiotics, biochemical studies, iminosugars, inhibitors, insect trehalase, trehalose, in vivo studies, mammalian trehalase, natural compounds, selectivity

## Abstract

In the era of green economy, trehalase inhibitors represent a valuable chance to develop non-toxic pesticides, being hydrophilic compounds that do not persist in the environment. The lesson on this topic that we learned from the past can be of great help in the research on new specific green pesticides. This review aims to describe the efforts made in the last 50 years in the evaluation of natural compounds and their analogues as trehalase inhibitors, in view of their potential use as insecticides and fungicides. Specifically, we analyzed trehalase inhibitors based on sugars and sugar mimics, focusing on those showing good inhibition properties towards insect trehalases. Despite their attractiveness as a target, up to now there are no trehalase inhibitors that have been developed as commercial insecticides. Although natural complex pseudo di- and trisaccharides were firstly studied to this aim, iminosugars look to be more promising, showing an excellent specificity profile towards insect trehalases. The results reported here represent an overview and a discussion of the best candidates which may lead to the development of an effective insecticide in the future.

## 1. Introduction

Insecticides and fungicides have played a fundamental role in raising the quality of our lives, not only for crop protection in agriculture, but also to avoid the spreading of harmful pests causing lethal human diseases, such as malaria. In the past, the non-restricted use of highly dangerous insecticides such as dichlorodiphenyltrichloroethane (DDT) has provoked negative effects in the environment and to mammals. DDT is a very toxic and persistent organic compound, its chemical stability provokes a long range transport into the environment and its non-hydrophilicity causes bioaccumulation in the tissues of animals and human beings. For these reasons, the US banned the use of DDT in 1972. However, DDT is still very efficient against malaria and other diseases caused by insects. In September 2006, the World Health Organization (WHO) declared its support for the indoor use of DDT in African countries where malaria remains a major health problem, citing that the benefits of the pesticide outweigh the health and environmental risks. The development of non-toxic, environmentally friendly insecticides and fungicides for human health and for crop protection is of great interest, especially for less developed countries affected by pandemic and starvation. The identification of a new target which is specific for insects and does not affect humans is therefore of particular relevance.

**Figure 1:**
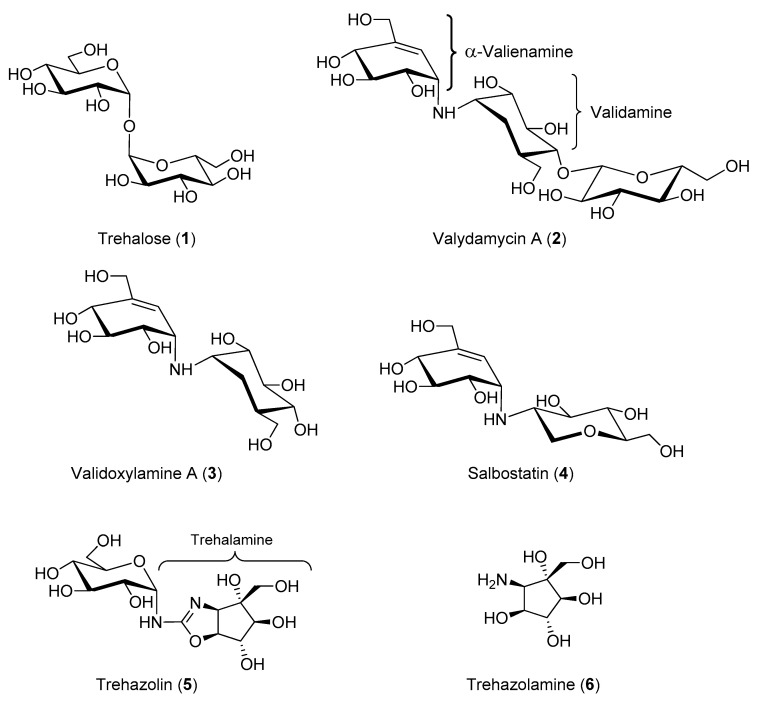
Structures of trehalose (**1**), the natural substrate of trehalase, and of some natural occurring carbohydrate- and carbocyclic-based trehalase inhibitors.

Trehalose (**1**, Figure 1) is a peculiar non-reducing disaccharide featured by the presence of two glucose units linked through an α,α-1,1-glycosidic linkage. This disaccharide is present in a wide variety of organisms, including yeast, fungi, bacteria, insects, some invertebrates, and lower and higher plants. However, trehalose is not found in mammals. In the 1970s, trehalose was merely regarded as a storage form of glucose for energy and/or for cellular components structure [1]. Since then, knowledge about the various functions of this simple disaccharide greatly expanded, and it is now evident that trehalose is much more than a simple storage compound [2], although its exact function in many organisms is still under investigation. In yeast and plants, trehalose is a signaling compound able to regulate certain metabolic pathways [3]. In addition, trehalose can act as a chemical “chaperone” [4] by stabilizing proteins in their native structure and thus preventing cellular damage from inactivation or denaturation caused by stress conditions such as desiccations, dehydration, heat, cold, and damage by oxygen radicals. When unicellular organisms are exposed to stress, they adapt by synthesizing huge amounts of trehalose, which contributes to retain cellular integrity by stabilizing protein structures in many different ways [5]. The equilibrium between trehalose storage and degradation needs to be finely tuned in response to different cellular states. In this regard, an important enzyme in trehalose metabolism is α-trehalase (EC 3.2.1.28), which has a regulatory role in controlling the levels of trehalose in cells by lowering its concentration once the stress is alleviated. Trehalose is indeed the main sugar circulating in the blood or hemolymph of most insects [6]. α-Trehalase (EC 3.2.1.28) is an inverting glycosidase [7] that promotes the conversion of trehalose into two molecules of glucose, which is vital for insect flight and essential for larvae resistance to stress factors [8].

The enzyme α-trehalase is also found in mammals both in the kidney brush border membranes [9] and in the intestinal villae membranes [10]. While its function in the kidney is not clear, the intestinal enzyme has the role of occasionally hydrolyzing ingested trehalose. Intolerance to fungi has been correlated with a deficit or a defect of the intestinal trehalase in mammals [2]. Trehalose is also an important component in fungal spores, where its hydrolysis plays a major role during early germination presumably serving as a source of glucose for energy [11].

Due to the biological relevance of trehalose and trehalose processing enzymes in pathological and physiological states, in particular for the important role of trehalose-derived glucose in larvae survival and development, trehalase inhibitors have been regarded in recent years as an interesting target for the identification of novel insecticides and fungicides. Therefore, in the last 50 years, several natural trehalose analogues and synthetic trehalose mimetics have been studied as potential fungicides and antibiotics. 

Nowadays, the search for green pesticides is an urgent issue and therefore, due to the presence of trehalase also in mammals, specificity towards the insect enzyme has become crucial for the development of drugs that are in principle safe for plants and mammals [12]. In addition, trehalase inhibitors are considered as valuable tools for studying the molecular physiology of trehalase function and sugar metabolism in insects [13].

In the past, several potent trehalase inhibitors were isolated from natural sources. These compounds include pseudodisaccharide structures and their glycosyl derivatives containing a sugar or a polyhydroxylated carbocycle (such as validamycins, validoxylamines, salbostatin and trehazolin, see Section 2). More recently, natural iminosugars were also reported as strong trehalase inhibitors (see Section 3). Iminosugars are nitrogenated glycomimetics with a nitrogen replacing the endocyclic oxygen of sugars, and widely known as glycosidase inhibitors [14].

This review aims to describe the efforts made in the last 50 years in the evaluation of natural compounds and their analogues as trehalase inhibitors, in view of their potential use as insecticides and fungicides. However, since isolation and purification of such compounds from natural source is very difficult and expensive, some of these structures have never been obtained in a chemically pure form. Therefore, considerable efforts have been devoted to their total synthesis and that of related analogues. In this regard, the syntheses of these compounds are highly challenging, due to the presence of several contiguous carbon stereocenters, which limited the number of synthetic approaches described, but in the same way resulted in the description of very elegant total syntheses. This review collects the efforts made by the researchers in the evaluation of carbohydrate-, carbocyclic-, and iminosugar-based compounds as trehalases inhibitors of different origins.

## 2. Carbohydrate- and Carbocyclic-Based Inhibitors

Between the 1970s and the 1980s, the pseudo-oligosaccharides complex of validamycins, a family of naturally occurring antibiotics, was isolated from *Streptomyces hygroscopicus subsp. limoneus* and characterized [15,16,17]. Validamycin A, the major component of the complex, rapidly emerged for its activity in controlling rice sheath blight caused by the phytopathogenic fungus *Rizochtonia solani* (*R. solani*) [15].

Validamycin A (**2**, Figure 1) possesses a unique pseudo-trisaccharide structure composed of an unsaturated carbasugar (α-valienamine), an amino carbasugar (validamine) and a β-d-glucose residue. The hydrolysis of the glycosidic bond affords the pseudo-disaccharide validoxylamine A (**3**, Figure 1), which showed an outstanding activity as trehalase inhibitor. Indeed, while validamycin A inhibited trehalase from *R. solani* with an IC_50_ = 72 μM, validoxylamine A resulted in a much more potent competitive inhibitor with a *K*_i_ = 1.9 nM (IC_50_ = 140 nM) (Table 1) [18]. For both **2** and **3** no significant activity was exhibited against cellulase, pectinases, α-amylase, and α- and β-glucosidases, showing an important selectivity for the trehalase enzyme. Interestingly, studies conducted to determine the uptake of these compounds into the mycelia of *R. solani* demonstrated that **2** was more readily taken up into the cells than its aglycone **3**. In addition, **2** significantly suppressed the degradation of intracellular trehalose at very low concentration (0.1 μg/mL) when incubated in mycelia of *R. solani*, thus showing remarkable in vivo activity as a trehalase inhibitor and explaining the origin of the antifungal properties. Taken together, these data suggest that validamycin A is efficiently transported into the mycelia and probably hydrolyzed therein by a β-glucosidase yielding validoxylamine A, which possesses a greater inhibitory activity [18]. The inhibitory activity of **2**, **3** and some structural analogues was tested on various trehalases from porcine intestine, rat intestine, rabbit kidney, baker’s yeast, *Mycobacterium smegmantis*, and *Spodoptera litura* (*S. litura*) insect [19]. Once more, **3** emerged as the best competitive inhibitor with *K*_i_ values from 0.31 μM (rat intestine) to 0.27 nM (baker’s yeast). The screening of validoxylamine A analogues demonstrated that the double bond in validoxylamine A is not essential to the activity as the configuration of the hydroxyl and hydroxymethyl groups is. Later reports confirmed that **3**, resembling trehalose in structure, showed potent and specific inhibitory activity towards trehalases in various organisms, from microorganisms to mammals [20,21,22] and in most cases the inhibition mechanism by validoxylamine A was competitive, with the exception of a non-competitive slow binding process reported in porcine kidney trehalase [22]. It is well known that insect hemolymph contains a high concentration of trehalose which is used as an energy source in various tissues. Administration of the potent trehalase inhibitor **3** to insects was thought to block energy metabolism leading to abnormal physiological function. Therefore, in the last 20 years, the effects of validoxylamine A on insects was investigated. Some studies reported only the inhibitory activity towards the trehalase enzyme extracted and purified from insects, such as the case of termites trehalase for which **3** showed to be a one order of magnitude more potent inhibitor than **2** (*K*_i_ = 3.2 μM vs. *K*_i_ = 402 μM) [23]. Other studies reported both the in vitro and in vivo inhibitory activity of validoxylamine A on insect trehalases, as well as its insecticidal activity. For example, the injection of validoxylamine A and its analogues to *S. litura* larvae showed that validoxylamine A was both the best inhibitor towards the *S. litura* trehalase (*K*_i_ = 43 nM, Table 1) and the most potent insecticide, providing 100% mortality at a dose of 10 μg/last instar larva [20]. Lethal activity exhibited by **3** in the common silkworm (*Bombyx mori, B. mori*) [24,25], in *Mamestra brassicae* (*M. brassicae*) [26,27] and in the American cockroach (*Periplaneta americana, P. americana*) [28,29] was also reported.

In the early 1990s the trehalase inhibitor salbostatin (**4**, Figure 1) was isolated from *Streptomyces albus* ATCC21838 and its full characterization revealed a pseudo-disaccharide structure, which can be considered a validoxylamine A analogue with a 1-deoxyglucosamine residue attached to the α-valienamine (Figure 1) [30]. A convenient total synthesis of salbostatin **4**, reported by Ogawa and coworkers, allowed screening of the inhibitory activity of **4** towards different trehalases [31]. Unfortunately, **4** showed a stronger inhibitory activity towards trehalase from porcine kidney (*K*_i_ = 0.18 μM) [30] than from silkworm (IC_50_ = 8.3 μM) [31] and this hampered its use as a non-toxic insecticide (Table 1).

**Figure 2:**
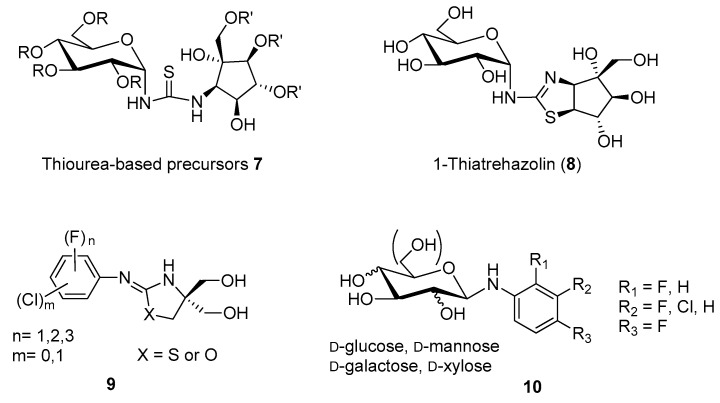
Structures of synthetic carbohydrate-, carbocyclic-, and heterocyclic-based trehalase inhibitors.

Isolated in 1991 from a culture broth of *Micromonospora* strain SANK 62390 [32,33], trehazolin (**5**, Figure 1) is a pseudo-disaccharide consisting of an α-d-glucopyranose moiety bonded to a unique aminocyclopentitol (trehazolamine **6**, Figure 1) through a fused 2-aminooxazoline ring. Although **5** can be considered a glucosylamine derivative, formally obtained by the reaction of trehalamine and d-glucose, this synthetic approach has never appeared in the literature. All reported syntheses employed thiourea derivative **7** (Figure 2) as precursor, which can be in turn obtained by diversely combining two subunits bearing an isothiocyanate moiety and an amino group, respectively. The synthesis of such fragments from different sugars and the subsequent strategies to access trehazolin (**5**) and analogues have been exhaustively reviewed by El Ashry and El Nemr in 2011 [34]. Compound **5** rapidly emerged as a slow, tight-binding inhibitor of silkworm trehalase [35], showing a remarkable selectivity with respect to other glycosidases (e.g., α- and β-glycosidases, maltase, isomaltase, sucrase, amyloglucosidase, etc.) and a reversible, competitive type of inhibition (*K*_i_ = 10 nM [35], IC_50_ = 49 nM [36], Table 1). The presence of the sugar moiety was proven essential for the inhibitory activity, being trehalamine much less potent in inhibiting silkworm trehalase (IC_50_ = 1 mM vs. IC_50_ = 52 nM) [35]. Compound **5** is a potent inhibitor of porcine trehalase as attested by two independent studies that reported IC_50_ = 16 nM [35] and IC_50_ = 19 nM (purified pig kidney trehalase) [21], respectively (Table 1). In order to investigate the structure–activity relationships with regard to the stereochemistry of the aminocyclitol moiety and that of the anomeric position, as well as the role played by hydroxyl functions, several analogues of **5** (epimers, β-anomer, deoxygenated aminocyclitol moiety congeners) were synthesized and tested.

Different studies confirmed that any structural modification at the pyranose or cyclopentitol moieties appreciably decreases **5** inhibitory potency, suggesting that its close resemblance to the substrate α,α-trehalose is responsible for the high and selective trehalase inhibitory activity [36,37,38]. This assumption was also extended to the modification of the oxazolidine ring obtained by replacing the oxygen atom with a sulfur atom. Indeed, Chiara and coworkers reported a highly stereoselective and efficient synthesis of 1-thiatreazolin **8** (Figure 2), which was proven to be a nanomolar, slow, tight-binding inhibitor of porcine trehalase, even though it is less potent than trehazolin itself (*K*_i_ = 30.4 nM vs. *K*_i_ = 2.1 nM, Table 1) [39]. However, 1-thiatrehazolin **8** is more stable than trehazolin **5** and for this reason in 2007 it was co-crystallized with Tre37A trehalase from *Escherichia coli* (*E. coli*), in the first report on the three-dimensional structure of a trehalase [40]. Davies and coworkers determined the enzyme structure in complex with validoxylamine A (**3**) and 1-thiatreazolin (**8**). Availability of these structures definitely proved the hypothesis earlier emerged from kinetic studies performed with porcine kidney trehalase in the presence of two types of competitive inhibitors [41], which suggested that the active centre of the enzyme may comprise two subsites, one for catalysis and one for recognition, acting separately on each glucose unit of trehalose. In particular, the complexes of Tre37A with **3** and **8** revealed the interactions in the −1 (“catalytic”) and +1 (“leaving-group”) subsites: the valienamine of **3** and the cyclopentane ring of **5** are placed in the −1 subsite, even though with different positions, while in the +1 subsite the pseudosugar ring of **3** and the glucose moiety of **8** lie in a nearly identical position, which promotes favorable hydrogen bonding with the same residues. In addition, the complexes of Tre37A with **3** and **8** strongly implicate Asp312 and Glu469 as the catalytic acid and base, confirming that the catalysis by trehalases occurs with inversion of the anomeric configuration [40].

In 1995, Nakajima and coworkers reported for the first time the pesticidal activity (namely, antifungal and insecticidal) of trehazolin **5** [42]. First, they investigated the protective and curative activities of **5** against plant pathogenic fungi and obtained remarkable results with the infection of rice seedlings caused by *R. solani*, which was completely inhibited by spraying a 100 ppm **5** solution one day after inoculation. This curative effect was ascribed to the activity of **5** as potent inhibitor of *R. solani* trehalase (IC_50_ = 66 nM, Table 1). It is worth noting that validamycin A was more effective in suppressing the growth of *R. solani* (curative effect at 7.5 ppm concentration) probably due to a more efficient uptake of this antibiotic into the mycelia of *R. solani* [18]. Due to the well-known inhibitory activity of **5** towards trehalase from silkworm (*B. mori*), the same authors investigated the insecticidal activity of this compound in *B. mori* larvae. Interestingly, 50 μg and 100 μg doses of **5** were insecticidal (7/10 and 10/10 injected larvae were killed, respectively), while no toxicity towards mice (intravenous administration at a dose of 100 mg/kg) was observed [42].

**Table 1:** Inhibition of insect trehalases from silkworm (*B. mori*) and tobacco cutworm (*S. litura*), fungi trehalase from *R. solani*, porcine kidney and porcine intestine trehalases by compounds **2**, **3**, **4**, **5** and **8**.

Compound	Silkworm (*B. mori*), IC_50_ (*K*_i_)	Tobacco Cutworm (*S. litura*), IC_50_ (*K*_i_)	*R. solani*, IC_50_ (*K*_i_)	Porcine Kidney, IC_50_ (*K*_i_)	Porcine Intestine, IC_50_ (*K*_i_)
**2**	n.d.	370 nM [19]	72 µM [18]	250 µM [21]	420 nM [19]
**3** ^a^	n.d.	48 nM [19] (43 nM) [20]	140 nM (1.9 nM) [18]	2.4 nM [21]	14 nM [19]
**4**	8.3 µM [31]	n.d.	n.d.	(0.18 µM) [30]	n.d.
**5** ^b,c^	49 nM [36] 52 nM [35] (10 nM) [35]	n.d.	66 nM [42]	15.5 nM [39] ^d^ (2.1 nM) [39]	n.d.
**8** ^a^	n.d.	n.d.	n.d.	83.0 nM [39] (30.4 nM) [39]	n.d.

^a^ Compounds **3** and **8** are potent inhibitors of Tre37A enzyme from *E. coli* with *K*_i_ values of 10 nM and 9 nM, respectively [40]; ^b^ IC_50_ values of 5.5 nM and 3.7 nM were also reported towards silkworm and porcine trehalase, respectively [32]; ^c^ **5** also inhibits locust flight muscle trehalase with *K*_i_ = 10 nM (in extracts) and *K*_i_ = 8 nM (on purified enzyme) [13]; ^d^ IC_50_ = 16 nM [35] and IC_50_ = 19 nM [21] were also reported for natural **5**; n.d.: not determined.

With the aim of clarifying the mechanism of toxicity exerted by trehalase inhibitors in insects, the effect of **5** on trehalase activity of locust flight muscles was investigated by Wegener and coworkers [13]. In vitro experiments showed that **5** acts as a competitive, tight binding inhibitor of locust flight muscle trehalase both when tested in extracts and on the purified enzyme (*K*_i_ = 10 nM vs. *K*_i_ = 8 nM) (Table 1). In vivo experiments revealed that **5** differentiates between an ‘overt’ and a ‘latent’ trehalase, the latter being catalytically inactive in vitro and probably derived from a trehalase form that is protected from inhibition by **5** in the intact insect. In addition, the insecticidal activity of **5** was proven: 50 μg injected into locusts completely and selectively blocked the overt form of muscle trehalase and killed 50% of the insects within 24 h. This study also demonstrated that **5** caused dramatic hypoglycemia: indeed, after injection of a 10 μg dose, glucose levels decreased by over 90% in 24 h. More interestingly, feeding glucose to the locusts fully neutralized the effect of a potentially lethal dose of **5**, indicating that hypertrehaloseanemia is not acutely toxic while lack of glucose causes organ failure, and that sufficient hemolymph glucose can be only generated from trehalose by trehalase. This and other similar studies [43] perfectly show that trehalase inhibitors are valuable tools for studying the molecular physiology of trehalase function and sugar metabolism in insects.

Aiming at developing artificial synthetic inhibitors of trehalase with simpler structures than the natural compounds **3** and **5**, Qian and co-workers prepared a series of fluorine-containing arylaminooxazo(thiazo)lidines **9** [44] and fluorinated *N*-arylglycosylamines **10** [45], respectively (Figure 2). Although compounds **9** were moderate porcine trehalase inhibitors (best inhibitor: IC_50_ = 31.6 μM), they were expected to increase the hydrophobicity and the penetrating ability in vivo. Indeed, some compounds of the series showed remarkable larvicidal activity and inhibition action on insect flight, when tested on fruit fly *Drosophila melanogaster* at 200 ppm concentration. Conversely, compounds **10**, designed to improve the antifungal activity in vivo with respect to validoxylamine A (**3**), failed in accomplishing this task toward *R. solani*, showing 133-fold lower antifungal activity as compared to validamycin A **2**.

## 3. Iminosugar-Based Inhibitors

Natural iminosugars are classified into five structural classes: polyhydroxylated pyrrolidines, piperidines, indolizidines, pyrrolizidines, and nortropanes [14]. For clarity, the discussion on the trehalase inhibitory activity of iminosugars has been divided based on their monocyclic or bicyclic structure.

### 3.1. Monocyclic Iminosugars and Their Derivatives

Although the inhibitory activity of iminosugars towards glycosidases was well documented from the 1970s, only in the early 1980s was the first evidence of the trehalase inhibition by monocyclic iminosugars emerged and the relationship between structure and selectivity towards trehalase over other glycosidases investigated.

**Figure 3:**
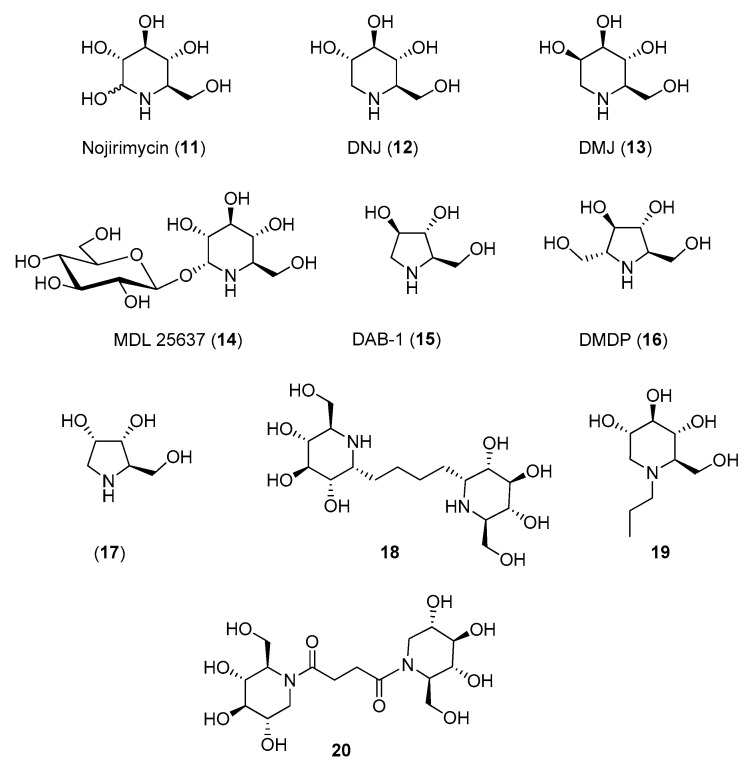
Structures of monocyclic iminosugar-based trehalase inhibitors and their derivatives.

The polyhydroxy piperidine family was tested first. Nojirimycin (**11**, Figure 3), known to be a more potent inhibitor of β-glucosidases than deoxynojirimycin (DNJ, **12**) [46,47] showed no inhibition towards insect trehalase [48], while DNJ (isolated from *Streptomyces lavendulae*) was reported to inhibit trehalase from *Chaetomium aureum* and from rabbit [49]. However, in 1994 Asano and coworkers reported that **12** strongly inhibited also α-glucosidases (IC_50_ = 0.3–0.4 μM) as well as porcine kidney trehalase (IC_50_ = 41 μM) [50], demonstrating its scarce selectivity. A better selectivity was observed for the d-mannose analogue of DNJ, deoxymannonojirimycin (DMJ, **13**), which showed an IC_50_ = 55 μM towards insect trehalase [48] and no inhibition towards mammalian trehalase (Table 2) [50]. Finally, the α-homonojirimycin-7-*O*-β-d-glucopyranoside (MDL 25637, **14**, Figure 3), whose synthesis was reported by Liu and coworkers in 1989 [51], showed an outstanding inhibitory activity towards porcine kidney trehalase (*K*_i_ = 3 nM) [22], suggesting that the presence of a glucose unit strongly enhances the affinity with the enzyme active site.

Regarding pyrrolidine iminosugars, 1,4-dideoxy-1,4-imino-d-arabinitol (DAB-1, **15**) was a good inhibitor of mammalian trehalase (IC_50_ = 4.8 μM towards porcine kidney trehalase). However, it was scarcely selective, showing good inhibitory activity also towards α-glucosidases (IC_50_ = 5.8–100 μM) and α-mannosidases (IC_50_ = 46–110 μM) [50]. In addition, its analogue DMDP (**16**), inhibited bacteria and insect trehalases (IC_50_ = 0.35 μM towards *Corynebacterium* sp. and IC_50_ = 10 μM towards *Plutella xylostella*) but did not inhibit porcine kidney trehalase (no inhibition at 1 mM) (Table 2) [52].

The relevant role played by the iminosugar moiety in trehalase inhibition was demonstrated by Bini and coworkers: a series of pseudo-disaccharide structures was synthesized by cross-metathesis (CM) reactions between suitably functionalized piperidine (namely nojirimycin **11**) or pyrrolidine (namely DAB-1 **15** and 1,4-dideoxy-1,4-imino-d-ribitol **17**, Figure 3) iminosugars and preliminary assays toward commercial porcine trehalase were reported [53]. While very small inhibition was observed with pyrrolidine-based dimers, a good inhibition was detected with nojirimycin dimer **18** (Figure 3), which showed *K*_i_ = 44 μM. Even more interestingly, reference glucose dimer did not show any inhibition at a concentration of 1 mM, highlighting that the nitrogen atom is essential for inhibition.

More recently, the inhibition of a membrane-bound trehalase from the larvae of the midge *Chironomus riparius* (*C. riparius*) was investigated with the aim of identifying differences between midge trehalase and mammalian trehalase [54]. The membrane-bound isoform of trehalase (trehalase-2) is believed to be the latent (inactive) form, more abundant in larvae, which is activated at the beginning of the prepupal period, thus representing a very interesting target. Forcella and coworkers reported for the first time the isolation and purification of the trehalase-2 isoform from *C. riparius* and demonstrated that this enzyme is highly specific for trehalose, with an affinity about 5-fold higher when compared to the mammalian trehalase. DNJ showed a competitive inhibition both towards *C. riparius* and porcine kidney trehalase (with IC_50_ = 2.83 μM and IC_50_ = 5.96 μM, respectively, Table 2), but there was significant dissimilarity in the kinetic behavior between the two enzymes. All these data, supporting the idea that the catalytic sites of trehalases from porcine kidney and insects have different recognition requirements, prompted the design of new DNJ and DAB-1 derivatives, aimed at enhancing the inhibitor specificity.

For example, the introduction of a propyl chain on the endocyclic nitrogen of DNJ (**19**, Figure 3) caused a slight decrease of activity with respect to DNJ (IC_50_ = 9.7 μM vs. IC_50_ = 2.8 μM towards *C. riparius* trehalase) but imparted a 10-fold selectivity towards the insect trehalase (IC_50_ = 109 μM vs. IC_50_ = 5.96 μM towards porcine kidney trehalase) (Table 2) [55]. A good degree of selectivity (3–7 times) was obtained also with *N*-bridged DNJ dimers, in which the two iminosugar units are linked through 2-, 3-, or 4-carbon atoms amidic spacers. In particular, dimer **20** (Figure 3) was 7-fold more active towards insect trehalase and thus more specific than DNJ (only twice as active on insect trehalase) (Table 2) [56]. More recently, a series of DNJ derivatives presenting thiolated or unsaturated *N*-alkyl chains of various lengths were synthesized [57]. Although they potently inhibited insect and mammalian trehalases with inhibition in the low micromolar range (IC_50_ values ranging from 0.27 μM to 6.94 μM), no selectivity toward insect trehalases was observed.

**Table 2:** Inhibition of *C. Riparius* and porcine kidney trehalases by compounds **12**, **13**, **15**, **16**, **19**, and **20**.

Compound	*C. riparius*, IC_50_ (*K*_i_)	Porcine Kidney, IC_50_ (*K*_i_)	Selectivity ^a^
**12**	2.83 μM [54] (1.39 μM) [54]	5.96 μM [54] ^b^ (2.98 μM) [54]	
**13**	55 μM [48]	n.i. [50]	18.2
**15**	19 μM [54] (9.3 μM) [54]	4.8 μM [50] ^c^ (5.3 μM) [54]	-
**16**	10 μM [52] ^d^	n.i. [52]	100
**19**	9.7 μM [55]	109 μM [55]	11.2
**20**	11 μM [56]	76 μM [56]	6.9

^a^ Selectivity is the ratio between the IC_50_ value against porcine kidney trehalase and the IC_50_ value against *C. riparius* trehalase; ^b^ IC_50_ = 41 μM [50] and IC_50_ = 43 μM [21] have been previously reported; ^c^ IC_50_ = 10.6 μM was also reported [54]; ^d^ IC_50_ reported towards insect trehalase from *Plutella xylostella* instead of *C. riparius*; n.i.: no inhibition at 1 mM.

Surprisingly, only one report appeared on the insecticidal activity of monocyclic iminosugars. In 2007, Hirayama and coworkers, inspired by the fact that mulberry latex contains very high concentrations of DAB-1 and DNJ as a defense against herbivory insects [58], added these inhibitors to the diet of *Samia ricini* (*S. ricini*) larvae and observed a significant increase in the hemolymph trehalose concentrations (trehalose concentration was approximately 50% higher than that in larvae fed on the standard diet) [59]. This effect, although interesting, is lower than the one observed in various other insect species with validoxylamine A (**3**), which is a strictly trehalase-specific inhibitor.

### 3.2. Bicyclic Iminosugars and Their Glycosyl Derivatives

The tropane alkaloids are bridged bicyclic amines combining a five-membered ring (pyrrolidine) and a six membered-ring (piperidine), with the nitrogen atom on the bridge. The tropane structure is also found in important medicinal alkaloids such as cocaine and atropine. These alkaloids are named calystegines and, due to their structural similarities with monocyclic iminosugars (such as DNJ, **12**), behave as glycosidase inhibitors.

**Figure 4:**
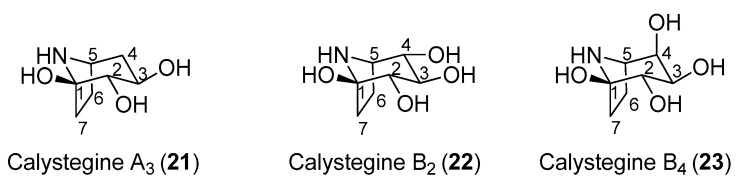
Structures of calystegines A_3_, B_2_, and B_4_.

In 1996, Asano and coworkers isolated several tropane alkaloids from the extracts of *Scopolia japonica*, a plant of the Solanaceae family [60]. A careful structural determination allowed the identification of a new compound, which was named as calystegine B_4_ (**23**, Figure 4) in addition to other known calystegines. Interestingly, the calystegine A_3_ (**21**), B_2_ (**22**), and B_4_ (**23**) (Figure 4) showed a remarkable and similar activity toward rat intestinal trehalase (IC_50_ values of 12.0, 9.0, and 9.8 μM, respectively). This indicated that the presence of a hydroxy group and the stereochemistry at C-4 of calystegines had no significant effect on inhibitory activity towards rat small intestinal trehalase. The three calystegines—**21**, **22**, and **23**—were also active towards pig kidney trehalase, with IC_50_ values of 13.0, 10.0, and 4.8 μM, respectively. These compounds were also tested towards non-mammalian trehalases of different origins. Calystegines B_2_ (**22**) and B_4_ (**23**) had very weak activities towards the enzyme from the pathogenic fungus *R. solani* (IC_50_ values of 700 and 540 μM, respectively), and none of the calystegines exhibited any appreciable inhibition toward baker’s yeast trehalase. More interestingly, calystegines **21**, **22**, and **23** exhibited good inhibitory activities toward the last instar larvae midgut trehalases form *B. mori* and *S. litura* (IC_50_ in a 19–50 μM range). Among the three calystegines shown in Figure 4, calystegine B_4_ (**23**) was more effective towards pig kidney trehalase than towards rat intestinal trehalase (IC_50_ = 4.8 μM vs. IC_50_ = 9.8 μM) and showed competitive inhibition with a *K*_i_ value of 1.2 μM. There are very few total syntheses of calystegine B_4_, which is due to the structural complexity of this compound [61,62,63].

**Figure 5:**
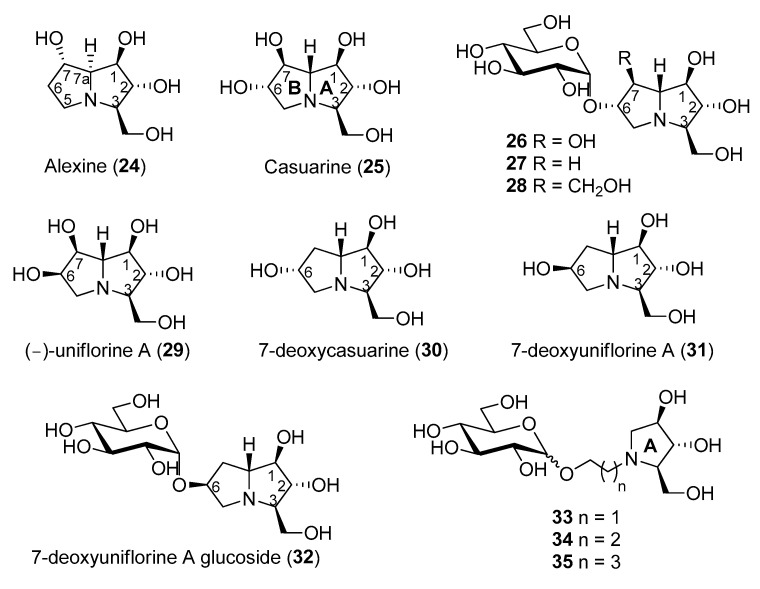
Structures of bicyclic or glycosylated iminosugar-based trehalase inhibitors.

Pyrrolizidines are bicyclic iminosugars bearing two fused pyrrolidine rings and a bridgehead nitrogen atom. Alexine (**24**, Figure 5) was isolated in 1988 from the pods of the legume *Alexa leiopetala* [64], and, differently from the previously known and broad class of pyrrolizidine alkaloids bearing a carbon substituent at C-1 [65,66], represented the first example of a pyrrolizidine alkaloid with a carbon substituent at C-3. Casuarine (**25**, Figure 5), a highly oxygenated pyrrolizidine, was isolated from the bark of *Casuarina equisetifolia* L. (Casuarinaceae) and from the leaves of *Eugenia jambolana Lam.* (Myrtaceae) [67], two plants widely employed in phytomedicine for their therapeutic action [68,69,70,71] together with its 6-*O*-α-d-glucopyranoside (**26**) [72]. During a study aimed at the structural elucidation of australine and related alkaloids, Asano and coworkers found that alexine (**24**) was a moderate inhibitor of porcine kidney trehalase (IC_50_ = 55 μM) [73]. In the same work, casuarine (**25**) was reported to inhibit this enzyme quite strongly (IC_50_ = 12 μM), and its 6-*O*-α-d-glucopyranoside (**26**) was an even more potent inhibitor. The glucoside **26**, indeed, inhibited porcine kidney trehalase with an IC_50_ = 340 nM and in a competitive manner, with a *K*_i_ value of 18 nM. However, the compound isolated from natural source showed a purity of about 60%, thus, presumably, the pure compound was expected to have a lower *K*_i_. Other examples of glycosyl iminosugars were later isolated from natural sources, indicating that their occurrence in nature is not so uncommon [74].

The total synthesis of these pyrrolizidine alkaloids is quite challenging, since they bear several contiguous and well defined stereocenters. The first asymmetric synthesis of (+)-alexine (**24**) was accomplished by Yoda and co-workers in 2000 through synthetic elaboration of a key lactam derived from 2,3,5-tri-*O*-benzyl-β-arabinofuranose [75]. Since then, several other total syntheses of (+)-alexine (**24**) were reported [76,77,78,79].

Regarding casuarine (**25**), its first total synthesis dates back to 1999 and was reported by Denmark and co-workers, who employed a tandem [4 + 2]/[3 + 2] nitroalkene cycloaddition as the key step of the strategy, which involved a nitroalkene, a chiral vinyl ether and a vinyl silane as starting materials [80,81]. Later on, (+)-casuarine (**25**) was synthesized by other research groups starting from carbohydrate derivatives [82,83,84]. Goti and coworkers developed a synthetic strategy aimed at the total synthesis of (+)-casuarine (**25**), based on a stereoselective carbohydrate-based nitrone cycloaddition with a proper alkene [85]. The key step of the synthesis involved a Tamao–Fleming oxidation for the correct installation of the OH group at C-7. The same strategy also allowed the first total synthesis of its 6-*O*-α-glucoside (**26**), through a selective α-glucosylation of a glucosyl donor with a pyrrolizidine acceptor bearing a non-protected OH group at C-6. The use of different alkenes allowed the synthesis of other casuarine derivatives and their biological evaluation towards a wide range of glycosidases [86]. The total synthesis of **26** allowed to obtain this compound for the first time in a chemically pure form. Two novel casuarine-6-*O*-α-d-glucoside analogues **27** and **28** (Figure 5) were also synthesized by Cardona and coworkers, by means of a similar selective α-glucosylation of a glucosyl donor with pyrrolizidine acceptors bearing no substitution or the CH_2_OH substitution at C-7, respectively [87]. The activity of this set of compounds was tested towards trehalases of different origins, namely porcine kidney, *E. coli* Tre37A [40] and *C. riparius* trehalases (Table 3) [54].

It was found that (+)-casuarine (**25**) was a strong inhibitor of both *E. coli* Tre37A and *C. riparius* trehalases (*K*_i_ = 17 μM and 0.12 μM, respectively). However, its 6-*O*-α-d-glucoside **26** was around 1000-fold more active (*K*_i_ = 12 nM and 0.66 nM, respectively), thus proving to be one of the most potent trehalase inhibitors described to date. The potency of glycosides **26**, **27**, and **28** towards the three trehalases reported in Table 3 showed a similar trend, demonstrating that the substitution at the C-7 position of the pyrrolizidine portion had a significant effect on the inhibition: the OH group at C-7 (in **26**) was preferential to a hydrogen atom (in **27**), and both were more potent than a CH_2_OH group (as in **28**). However, in terms of specificity of inhibition, compounds **27** and **28** were more interesting, since they showed to be more selective towards the insect *C. riparius* trehalase over the mammalian enzyme. In particular, glucoside **27** displayed 6.3-fold selectivity, while glucoside **28** was more than 60-fold selective towards the *C. riparius* over the porcine kidney trehalase [87].

Tre37A enzyme has a buried cavity with two subsites. The −1 subsite is the catalytic site and it is specific for the substrate, while the +1 subsite is specific for the glucoside leaving group [40]. X-ray analysis of compound **26** in complex with the enzyme Tre37A was collected to 1.9 Å resolution [85] and revealed that the casuarine portion in **26** was bound to the −1 subsite of Tre37A, while the glucose moiety of **26** was placed in the +1 leaving group subsite. As the binding of casuarine (**25**) leaves free one of the two subsites of Tre37A, fewer interactions can form, and this rationalizes the lower affinity of ‘monosaccharide-like’ inhibitors with respect to ‘disaccharide-like’ ones. Interestingly, a sulfate group was co-crystallized together with **26** and formed hydrogen bonds with the OH at C-6 position of the sugar portion. More or less the same situation was found for the complex of **28** with Tre37A, which was collected to 2.1 Å resolution [87]. The pyrrolizidine portion was found in the -1 catalytic site with the CH_2_OH group forming key hydrogen bonds interaction, and again a sulfate group was co-crystallized and formed hydrogen bonds with near residues.

(−)-Uniflorine A (6-*epi*-casuarine, **29**, Figure 5) was isolated in 2000 from the leaves of the tree *Eugenia uniflora* L. (Myrtaceae). This plant, widely distributed in South America, was used to prepare infusions used in folk medicine in antidiabetic preparations [88]. In 2004, the group of Pyne and co-workers proved, through the total synthesis of putative (−)-Uniflorine A, that the originally proposed structure (a polyhydroxylated indolizidine alkaloid) was not correct, thus demonstrating again the importance of total synthesis [89]. The structural reassignment was accomplished through the synthesis of its enantiomer (+)-uniflorine A [90]. Goti and coworkers accomplished the first total synthesis of **29** exploiting a similar strategy that allowed also the synthesis of (+)-casuarine (**25**) [91]. More recently, (−)-Uniflorine A was synthesized by SmI_2_-mediated radical cross-coupling [92].

As shown in Table 4, **29** and its 7-deoxy analogue (7-deoxyuniflorine A, **31**, Figure 5) showed remarkable inhibitory properties against *C. riparius* trehalase (IC_50_ = 177 and 175 nM, respectively) in spite of not bearing an additional glucosyl moiety at C-6. More importantly, they both showed excellent selectivity (>5000) towards the insect trehalase. This finding suggested that the stereochemistry at position 6 was crucial to impart such selectivity and that the presence of an additional glucosyl moiety at C-6 was not essential. Compound **30**, with the opposite stereochemistry at C-6, was active only in the low micromolar range (IC_50_ = 1.22 μM) toward the insect enzyme, while the glucoside derivative **27** was more active (IC_50_ = 44 nM) but less selective (Table 4) [93]. However, preliminary tests in vivo of compound **31** on *S. littoralis* larvae failed.

With the aim of synthesizing a more active glucoside derivative, Matassini and Cardona embarked with the total synthesis of glucoside **32**, which bears the same stereochemistry at C-6 of **29** and **31** [94]. Quite disappointingly, this compound showed activity only in the micromolar range (IC_50_ = 29.49 μM towards *C. riparius*), thus showing that a pyrrolizidine with this configuration is not able to place the glucosyl moiety within the enzyme cavity with favorable orientations. In order to reduce the overall number of synthetic steps, they also synthesized a series of simple and more flexible disaccharide mimetics 33–35 (Figure 5) with a pyrrolidine nucleus instead of the pyrrolizidine core, different spacers connecting the sugar and the iminosugar portion, and both configurations at the glucosyl moiety. Among the compounds synthesized, only compound **33 β** was active in the low micromolar range (IC_50_ = 0.784 μM towards *C. riparius,* Table 4) and showed good selectivity towards the insect enzyme.

## 4. Conclusions

The development of non-toxic environmentally friendly insecticides and fungicides for human health and for crop protection is of particular relevance, especially for less developed countries. Trehalase (EC3.2.1.28) has been identified in this regard, as a new target which is specific for insects and does not affect humans. Trehalase hydrolyses trehalose to two glucose units, a process which is essential to the life functions of several organisms, in particular fungi and insects, but does not affect vertebrates, who do not depend on the hydrolysis of this sugar. Thus, at least in principle, trehalase inhibitors were excellent candidates as new ‘green and safe insecticides’ to be used for protecting humans from diseases brought by insects and for crop protection.

Despite their attractiveness as a target, up to now there are no trehalase inhibitors that have been developed as commercial insecticides. This could be ascribed to a non-favorable plant uptake due to the high hydrophilicity of these compounds. Indeed, validamycin A, the only trehalase inhibitor that has been commercialized from the late 1960s (by Takeda Chemical Industries) for its curative effect toward the pathogen *R. solani*, was shown to behave as a pro-drug, being able to form the active pseudo-disaccharide trehalase inhibitor validoxylamine A in situ.

However, carbohydrate and carbocyclic based inhibitors with a pseudo-disaccharide structure (such as validoxylamine A, **3**) are very potent trehalase inhibitors but they are often not specific towards insects over the mammalian enzymes, and therefore cannot be considered as non-toxic. An effective alternative seems to be offered by the natural pseudo-disaccharide trehazolin (**5**), which shows remarkable insecticidal activity in larvae from different species, although it is not toxic towards mice. However, trehazolin strongly inhibits porcine kidney trehalase, the general model for the mammalian enzyme, thus suggesting that a deeper comprehension of the inhibition profile is necessary to develop safer inhibitors.

Indeed, when designing an inhibitor, the specificity is a very significant issue to be considered: the design of an effective insecticide which is able to target a given trehalase would require to be safe for plants and mammals, and if possible, also for insects which are of benefit in nature. In this regard, some iminosugar-based inhibitors showed an excellent specificity profile towards insect *C. riparius* trehalase. In particular, glucosyl derivatives of pyrrolizidine iminosugars showed excellent inhibitory activities toward this insect enzyme but, more importantly, it came out that the presence of an additional glucosyl moiety is not essential for selectively targeting the insect trehalase. In our opinion, the simplest pyrrolizidines **29** and **31** showing the highest selectivity towards the insect trehalases are among the most attractive hit compounds that deserve further studies in this direction. Although preliminary in vivo experiments did not give the desired effect, we deem that further studies are needed, in particular aiming at the transformation of the hit-compounds into pro-drugs with more favorable physico-chemical properties that will improve plant-uptake.

## Figures and Tables

**Table 3:** Inhibition of porcine kidney, Tre37A and *C. riparius* trehalases by compounds **25**, **26**, **27**, and **28**.

Compound	Porcine Kidney, *K*_i_	*E. coli* Tre37A, *K*_i_	*C. riparius*, *K*_i_
**25**	12 μM [73] ^a^	17 μM [85]	0.12 μM [54]
**26**	11 nM [87]	12 nM [87]	0.66 nM [87]
**27**	138 nM [87]	86 nM [87]	22 nM [87]
**28**	>10 μM [87]	2.8 μM [87]	157 nM [87]

^a^ Expressed as IC_50_.

**Table 4:** Inhibition (IC_50_) of porcine kidney and *C. riparius* trehalases by compounds **29**, **30**, **31**, **27**, **32**, **33**, **34**, nd **35**.

Compound	Porcine Kidney, IC_50_	*C. riparius*, IC_50_	Selectivity ^a^
**29**	>1 mM [93]	177 ± 18 nM [93]	>5649
**30**	20.6 ± 2.2 μM [93]	1.22 ± 0.08 μM [93]	17
**31**	>1 mM [93]	175 ± 12 nM [93]	>5714
**27**	479 ± 45 nM [93]	44 ± 1.0 nM [93]	10
**32**	190.60 ± 34.14 μM [94]	29.49 ± 7.26 μM [94]	6
**33 α,β**	7.67 ± 3.91 μM [94]	2.30 ± 0.13 μM [94]	3
**33 α**	27.64 ± 5.35 μM [94]	9.36 ± 1.49 μM [94]	3
**33 β**	5.84 ± 0.26 μM [94]	0.784 ± 0.059 μM [94]	7
**34 α,β**	n.d.	>1 mM	-
**35 α,β**	n.d.	>1 mM	-

^a^ Selectivity is the ratio between the IC_50_ value against porcine kidney trehalase and the IC_50_ value against *C. riparius* trehalase; n.d.: not determined.
